# Mineral Oil in Peptic Ulcer

**Published:** 1917-02

**Authors:** 


**Mineral Oil in Peptic Ulcer.** K. M. Ferguson, Va. Med. Semi-monthly, Sept. 8, 1916, quoted in Critic and Guide, Jan., mentions this as a new treatment, the action being largely to favor the elimination of toxins by the laxative effect. The editor began to use mineral oil for gastro-intestinal ulcerations about 1897 and has, since then, employed it with bismuth, etc., almost as a routine. The idea is, therefore, by no means new. In addition to the eliminant action, that of local protection must also be considered. This treatment, however, is somewhat contraindicated in acute peptic ulcer, though rather by apprehension of the possible effects of introducing any medicament into the stomach, than as a matter of experience. It is especially indicated in various other forms of ulcer, degenerative, etc.

